# Self-Organogenesis from 2D Micropatterns to 3D Biomimetic Biliary Trees

**DOI:** 10.3390/bioengineering8080112

**Published:** 2021-08-05

**Authors:** Emilie Gontran, Lorena Loarca, Cyrille El Kassis, Latifa Bouzhir, Dmitry Ayollo, Elsa Mazari-Arrighi, Alexandra Fuchs, Pascale Dupuis-Williams

**Affiliations:** 1 Physiopathogenèse et Traitement des Maladies du Foie, Université Paris-Saclay, Inserm, F-94800 Villejuif, France; Emilie.GONTRAN@gustaveroussy.fr (E.G.); cyrille.el-kassis@inserm.fr (C.E.K.); latifa.bouzhir@universite-paris-saclay.fr (L.B.); 2 INSERM U-1279, Gustave Roussy, F-94805 Villejuif, France; 3 INSERM, Institut Universitaire d’Hematologie, Université de Paris, U976 HIPI, F-75006 Paris, France; dmitry.ayollo@gmail.com (D.A.); elsa.mazari@gmail.com (E.M.-A.); Alexandra.FUCHS@cea.fr (A.F.); 4 AP-HP, Hôpital Saint-Louis, 1 Avenue Vellefaux, F-75010 Paris, France; 5 CEA, IRIG, F-38000 Grenoble, France; 6 ESPCI Paris, Université PSL, F-75005 Paris, France

**Keywords:** intrahepatic, biliary duct, micropattern, cholangiocyte, self-organogenesis

## Abstract

Background and Aims: Globally, liver diseases account for 2 million deaths per year. For those with advanced liver disease the only curative approach is liver transplantation. However, less than 10% of those in need get a liver transplant due to limited organ availability. To circumvent this challenge, there has been a great focus in generating a bioengineered liver. Despite its essential role in liver functions, a functional biliary system has not yet been developed. In this framework, exploration of epithelial cell self-organogenesis and microengineering-driven geometrical cell confinement allow to envision the bioengineering of a functional biomimetic intrahepatic biliary tract. Approach: three-dimensional (3D) bile ducts were built in vitro by restricting cell adhesion to two-dimensional (2D) patterns to guide cell self-organization. Tree shapes mimicking the configuration of the human biliary system were micropatterned on glass slides, restricting cell attachment to these areas. Different tree geometries and culture conditions were explored to stimulate self-organogenesis of normal rat cholangiocytes (NRCs) used as a biliary cell model, either alone or in co-culture with human umbilical endothelial cells (HUVECs). Results: Pre-seeding the micropatterns with HUVECs promoted luminogenesis with higher efficiency to yield functional branched biliary tubes. Lumen formation, apico-basal polarity, and preservation of the cholangiocyte phenotype were confirmed. Moreover, intact and functional biliary structures were detached from the micropatterns for further manipulation. Conclusion: This study presents physiologically relevant 3D biliary duct networks built in vitro from 2D micropatterns. This opens opportunities for investigating bile duct organogenesis, physiopathology, and drug testing.

**Highlights**

- Biliary ducts have been precluded from previous liver bioengineering studies.
- Bile ducts were generated from cholangiocytes self-organization on micropatterns.
- Co-culture with endothelial cells allowed the formation of millimeter long biliary networks with interconnected lumens.
- This is the first model of intrahepatic biliary ducts of defined geometry.

## 1. Introduction

Even though the biliary system plays a critical role in food digestion and liver detoxification [1], bioengineering of a functional biliary tree has not yet been successful. Several approaches have been described in an effort to bioengineer bile ducts: from spontaneous self-organization of rodent or human cholangiocytes, cholangiocytes from bile, and cholangiocyte-like cells differentiated from liver progenitor cells into functional spheroids and tube fragments in natural hydrogels or decellularized extracellular matrix (dECM) either as mono-cultures or co-cultures with other liver cells [2,3,4,5,6]; to the construction of biliary tubes from populated tubular constructs, or bioprinting in hepatic dECM and the development of functional biliary tube fragments in a microfluidic chip [7,8,9]. Moreover, the effects of the media flow on the hepatocyte phenotype and functions were investigated on a 3D hepatobiliary model [10]. However, most of these studies have focused on generating spheroid-like or monoaxial tube fragments [11,12,13,14]. Others have elegantly shown the formation of randomly organized biliary tree structures [15]. The role of branching angles and tube widths within the millimeter range (extra-hepatic bile ducts) were investigated in directing the growth of these structures [16].

Microengineered platforms with modifiable substrate chemistry are a tool to control cell geometry, cell arrangement, and cell adhesion over the surface. The idea is to restrict cell spreading and cell volume, mimicking to some extent physiological environments [17]. 2D and 3D micropatterning with substrates such as natural hydrogels [18], synthetic polymers [19], extracellular matrix components (ECM) [20], and growth factors [21] have been used in a wide range of geometries and designs including tube-like shapes and networks [18,20,22]. For instance, Hauser and colleagues generated functional tubes from renal progenitor cells and ureteric bud cells on agarose gel-micropatterns [18]. Notably, 3D tubes from kidney cells self-assembled on 2D micropatterns coated with different ECM components [20]. The role of ECM components, stiffness, and tube dimensions in luminogenesis and tubulogenesis was highlighted using this technology [20]. Moreover, tubulogenesis of endothelial cells on micropatterns revealed a key role for pericytes in tubular structure and lumen stability [22].

In the present study we used 2D micropatterning to generate 3D interconnected tubes mimicking the geometry of biliary trees. We integrated various microscopy and macroscopy techniques to characterize the 3D functional biliary trees. Micropatterns of physiologically relevant tree geometries of various angles and widths that range from 10 µm to 150 µm mimicking the dimensions of the distal part of the human intrahepatic biliary system were manufactured. Various culture conditions were explored, NRCs were used as a source of biliary epithelial cells, and either cultured alone or with HUVECs. Interestingly, endothelial cells appeared to be important for luminogenesis of functional 3D biliary networks. This report proves the formation of functional and polarized 3D biliary networks grown from 2D micropatterns. This simplified model might help to reveal some specific properties of cholangiocytes in biliary tree organogenesis, physiopathogenesis, and detoxification. Further work will focus on adapting a microfluidic system to our model to study the impact of flow and bile composition on bile epithelium homeostasis. Finally, this model will also pave the way for building a bioengineered liver, by adding gradually surrounding liver cells.

## 2. Results

### 2.1. Bile Duct Trees Formed by Self-Organization from Micropatterns

Cholangiocytes self-organize into cysts that consist of a monolayer surrounding a central lumen when embedded in a suitable hydrogel [23]. Based on this feature, previous experiments in our lab (not shown) showed that partial tubulogenesis could be induced by adding Matrigel to NRCs grown on adhesive rectangles, and for widths ranging from 10 to 200 microns.

Therefore, we cultured the cholangiocytes on tree-shaped micropatterns of the same dimensions, pre-seeded with endothelial cells, for their supportive function in the development of many epithelial tissues [24] and their critical role during embryogenesis of the biliary system [25,26].

**Figure 1:**
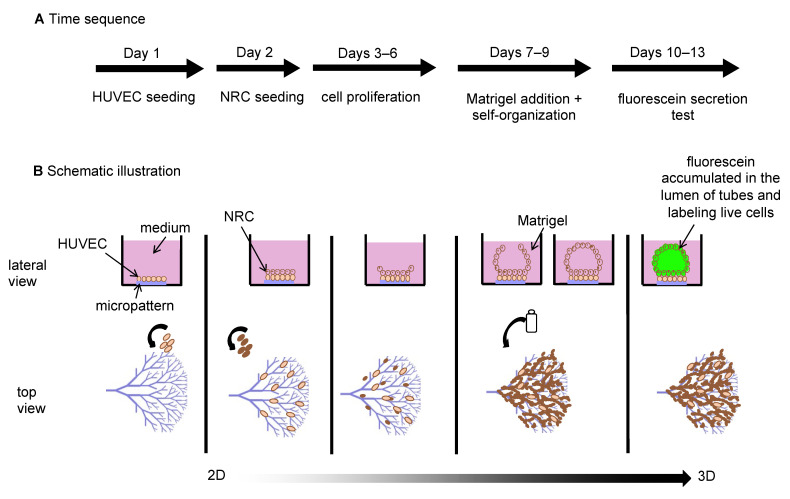
Experimental workflow of biliary tube engineering based on 2D micropatterning and 3D growth stimulation. (**A**) cell culture protocol: day 1 to day 6: cell seeding and cell proliferation in 2D on micropatterns, Matrigel addition, and self-organization from days 7 to day 10/13 in 3D. A fluorescein secretion test was performed on day 10/day 13 to reveal the formation of lumens. (**B**) schematic illustration of the lateral and top views of the experimental setup.

The experimental protocol was divided into three sequential steps: (1) cell seeding and proliferation in 2D from day 1 to day 6 (Figure 1A) until cells reached confluency filling the shape of the tree micropatterns; (2) 3D growth stimulation by adding Matrigel at day 7 to induce tubulogenesis in 3D from day 7 to day 10/day 13 (Figure 1B); (3) a fluorescein secretion test used to label live cells, revealing closed luminal spaces resulting from the export of fluorescein by the NRCs transporter proteins (Figure 1, Supplementary Figure S1, Supplementary Video S1).

### 2.2. Study of the Luminogenesis of Bile Duct Structures

**Figure 2:**
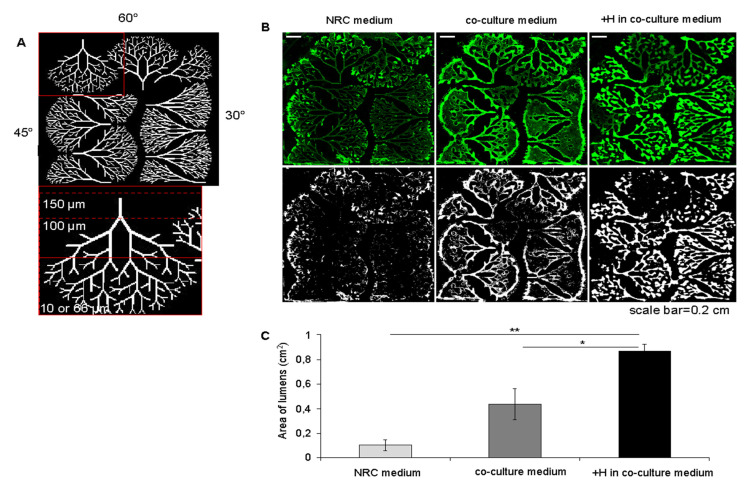
HUVECs enhance luminogenesis of 3D biliary networks. (**A**) photomask of the micropatterns showing physiologically relevant branch and angle dimensions. (**B**) fluorescein stained biliary network and corresponding binarized pictures illustrating the effect on tubulogenesis of pre-seeding HUVECs on micropatterns (scale bar = 0.2 cm). (**C**) luminal area quantification in NRC medium = NRCs alone cultured in NRC medium, co-culture medium = NRCs alone cultured in 50% NRC medium with 50% HUVEC medium, +H in co-culture medium = NRCs:HUVECs co-cultured in a 10:1 cell ratio in 50% NRC medium with 50% HUVEC medium, mean ± SEM, and N = 3 experiments per condition. One way ANOVA test with Tukey′s multiple comparisons test: NRC medium vs. +H in co-culture medium: *p*-value = 0.0018 < 0.01 **; co-culture medium vs. +H in co-culture *p*-value = 0.0273 < 0.05 *.

Micropatterns were fabricated with tree geometries by varying their branching angles: 30°, 45°, or 60°. All trees were designed with the same tree structure made of four regions of branch widths: 10, 66, 100, and 150 µm (Figure 2A). These micropatterns were designed to mimic the dimensions of the human distal biliary tree from the septal ducts (~100–300 µm diameter) to the intralobular ducts and ductules [27]. 

To determine the formation of closed luminal structures, 10-day-old biliary networks were incubated with fluorescein diacetate (FDA), which is hydrolyzed into the green fluorescent molecule fluorescein in live cells. In polarized cells, the multidrug resistance-associated protein 1 (MDR1) drives the export of fluorescein out through the apical side [27,28]. Thus, in tubular networks with closed luminal structures, fluorescein is retained in the lumen (Supplementary Video S1) and fluorescence intensity is higher than in incomplete luminal structures or 2D epithelia (Supplementary Figure S1). Based on this principle, the mean area of lumens was quantified over the micropatterns via stereoscopic microscopy analysis to assess the continuity of lumens (see Section 4).

Next, the formation of luminal structures was compared in the different conditions, NRC + HUVEC to controls i.e., NRCs alone in their culture medium, or NRCs in the co-culture medium in 10-day-old tubular structures. Seeding NRCs in their reference medium (Figure 2B: NRC medium), led to self-organized structures with limited luminogenesis (mean area of 0.1 ± 0.05 cm^2^), restricted to the tip of the trees, with some overlaps between the closest branches in the 30° and 45 ° trees. For NRCs alone in co-culture medium (Figure 2B: co-culture medium), the NRCs formed epithelia that extended out of the adhesive zones of the micropattern, between the closest branches, covering important proportions of small angle-trees. Considering that the co-culture medium sustains an overproliferation of 50% of the NRCs (Supplementary Figure S3), we hypothesized that the cell spreading out of the adhesive patterns results from an increase in cell proliferation. However, the lumens were also restricted to the end of the tree branches forming a narrow continuous space of 0.44 ± 0.13 cm^2^ delimiting the outline of the trees, where adhesive surfaces faced larger non adhesive areas. In the co-culture medium, the luminal area was over four-fold greater than in the NRC medium (Figure 2C), limited to the edges of the trees, and did not allow for tubulogenesis to take place on the internal branches. In the presence of HUVECs, the luminal networks expanded over the whole tree space, resulting in a mean luminal area of 0.87 ± 0.05 cm^2^ (Figure 2C: +H in co-culture medium), approximately two-fold greater than for NRCs alone in the co-culture medium).

In conclusion, although the increase in cell proliferation likely favors the extension of luminal areas, the pre-seeding of the patterns with HUVECs is instrumental in mediating the formation of a full luminal network with a geometry that is conditioned by that of the pattern. This suggests a role for HUVECs in delimiting the adhesive surfaces from the non-adhesive areas.

### 2.3. Characterization of Bile Duct Lumens

**Figure 3:**
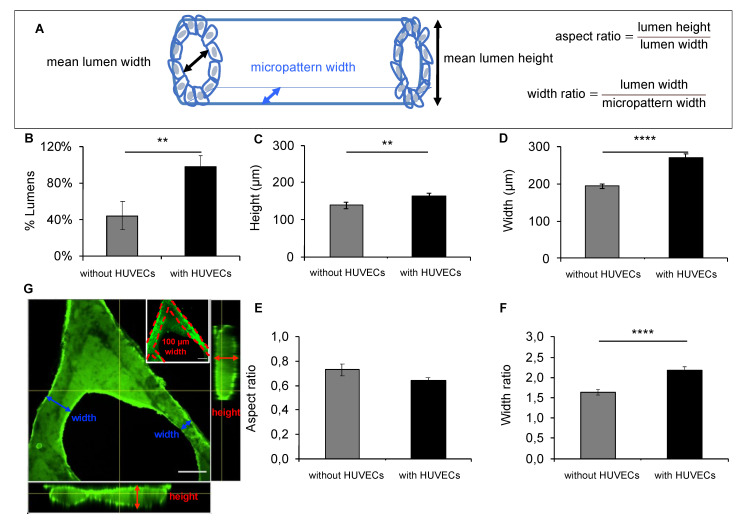
Impact of culture conditions on tubular geometry and frequency of lumen formation. (**A**) scheme depicting tube height and width, micropattern width, and aspect and width ratios formulas. Analysis of confocal pictures of fluorescein-stained day 10 tubular networks post-HUVEC seeding (with HUVECs, black bars) or NRC seeding (without HUVECs, gray bars) in the co-culture medium on 100 µm and 150 µm micropattern branches, *n* = 5 independent experiments and 65 and 34 confocal stacks were analyzed to calculate the mean in the presence or absence of HUVECs, respectively, (**B**) percentage of lumens ± SEM, (**C**) height ± SEM, (**D**) width ± SEM, (**E**) aspect ratio ± SEM, and (**F**) width ratio ± SEM, and (**G**) transversal projection (XY plane) of a biliary network structure secreting fluorescein into its lumen imaged in confocal and corresponding longitudinal views (XZ and YZ planes) formed on a 100 µm-branches micropattern. Scale bar = 200 µm. Mann–Whitney test: (**B**) *p*-value = 0.0079 < 0.01 **; (**C**) *p*-value = 0.0013 < 0.01 **; (**D**) *p*-value < 0.0001 ****; (**E**) *p*-value = 0.1382; not significant; (**F**) *p*-value < 0.0001 ****.

The geometry of 10-day-old tubular networks was further characterized via confocal microscopy after a fluorescein secretion test, which allows imaging of both cells and lumens, by preserving the cell and tissue architecture and dimensions (Section 4) (Supplementary Figures S1 and S2). Lumen occurrence and luminal tube height and width of 100 and 150 µm-width branches were assessed. In addition, to characterize the tubes′ 3D shape and to evaluate how accurate the luminal structure was with respect to the micropattern configuration, we calculated: (1) the aspect ratio of the tubes, defined by the ratio of their height over their width and (2) the ratio of the tube width over the width of the micropattern width (Figure 3A–G).

Next, we quantified the proportion of luminal structures formed at day 10 depending on the culture conditions: NRCs alone in the co-culture medium or NRCs with HUVECs in the co-culture medium (Figure 3B–F). Consistently, two-fold more lumens were formed when the micropatterns were pre-seeded with HUVECs (Figure 3B). The tubular networks were slightly taller (Figure 3C) and wider (Figure 3D) in the presence of HUVECs. Tubes formed in both culture conditions had a flattened shape evidenced by similar aspect ratios (Figure 3E). In both cases, the width ratios were above 1 meaning that the 3D structures spreaded out of the micropatterns. Tubes formed with HUVECs were two-fold wider than the micropatterns with a mean width ratio of 2.17 ± 0.09 (Figure 3F), as compared to 1.63 ± 0.06 (Figure 3F) without HUVECs.

The possible impact of the geometry of the micropatterns on the tubulogenesis was further investigated by classifying the measurements according to the tree angles (30°, 45°, or 60°) and the branch widths (Supplementary Figure S4). We confirmed that the increase in luminogenesis induced by the presence of HUVECs is stronger for the trees with smaller branching angles, in other words, when the epithelia are in a short distance to each other. The tubes′ heights and widths were also increased in the presence of HUVECs on the 150 µm branches and 60° trees. The aspect ratios show that the wider the tubes, the flatter they are, suggesting that the ECM in which they develop in 3D cannot provide the mechanical support to sustain a circular shape of the wider tubes.

In conclusion, the presence of endothelial cells favored the formation of larger tubes, but more importantly supported the continuous luminogenesis over longer distances. This allowed the development of interconnected tubular networks with a geometry that globally follows the one of the 2D pattern.

### 2.4. Bile Duct Structures Retain Apico-Basal Polarity with Preserved Cholangiocyte Phenotype

**Figure 4:**
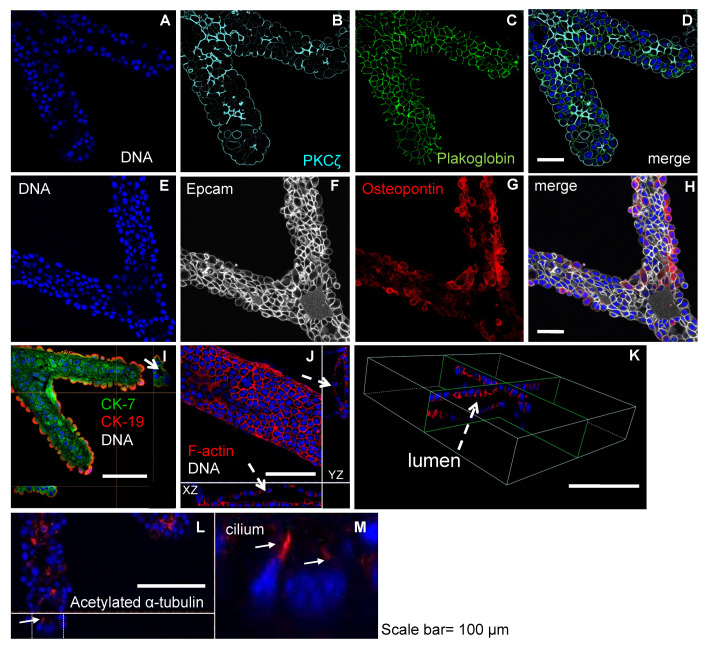
Tubular networks are polarized and retain the biliary phenotype. Confocal microscopy of immunofluorescence images of tubular networks at day 10 post-HUVEC seeding in NRCs:HUVEC 10:1 co-culture showing a single focal plane of (**A**) nuclei, (**B**) PKCζ, (**C**) plakoglobin, (**D**) merge, (**E**) nuclei, (**F**) Epcam, (**G**) osteopontin, and (**H**) merge. Orthogonal views of Z-stacks of (**I**) CK7-CK19-tubes (white arrow pointing at a lumen on the YZ plane), (**J**) F-actin 3D reconstruction showing the XZ and YZ orthogonal views of the inside of an F-actin labeled tree branch, (**K**) 3D orthogonal views of a single luminal structure (pointed as “lumen”) with nuclei staining and F-actin expressed at the apical face of cells, and (**L**) and (**M**) inset ortho views showing acetylated α-tubulin revealing the presence of primary cilia, scale bar = 100 μm.

Immunofluorescence analysis confirmed that the cholangiocytes in the trees retained apico-basolateral polarity with preserved cholangiocyte phenotype. Tubes were positive for the expression of apical proteins such as PKCζ, osteopontin, F-actin, and acetylated α-tubulin (Figure 4B,G,J–M), in addition to basolateral markers such as plakoglobin and epithelial adhesion molecule (Epcam) (Figure 4C,F). F-actin was found on the apical side of lumens and acetylated α-tubulin labeled primary cilium (Figure 4J–M) confirming the presence of well-polarized epithelial cells. Using orthogonal projections we confirmed that the biliary structures had a continuous lumen (Figure 4J,K). The networks also maintained expression levels of key cholangiocyte markers for bile ducts such as cytokeratin 7 (CK7) and cytokeratin 19 (CK19) (Figure 4I) [26,29]. These results suggest that the biliary network constructs were made of compact layers of cells retaining key epithelial and cholangiocyte features. This resulted in tight luminal structures with preserved secretion properties, as revealed by fluorescein secretion tests (Supplementary Videos S1 and S2).

### 2.5. Trees Can Be Detached and Remain Functional

**Figure 5:**
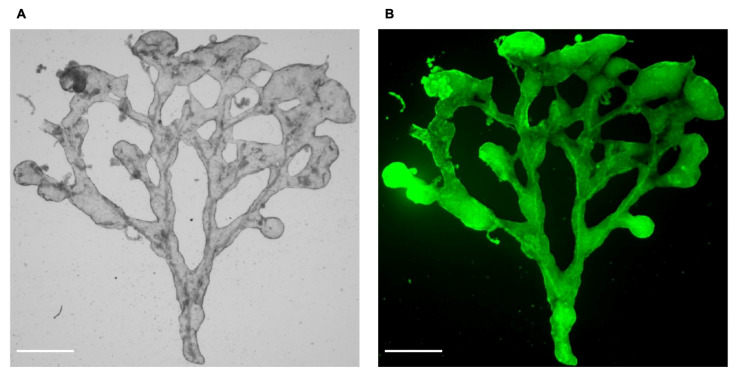
Biliary trees grown on micropatterns can be detached. Biliary tree from NRCs:HUVEC 10:1 co-culture detached from a 60° angle-micropattern, plated on Matrigel in the co-culture medium. (**A**) brightfield and (**B**) fluorescence stereomicroscopy images after a fluorescein secretion test showing the luminal tubular network, scale bar = 1 mm.

Interestingly, trees could be easily detached from micropatterned glass coverslips (see Section 4). Those trees could be transferred into Matrigel or kept in culture medium. The integrity of their architecture and functionality was assessed by stereomicroscopy imaging and FDA secretion (Figure 5 and Supplementary Video S2). The time lapse imaging of the FDA secretion illustrates the progressive accumulation of fluorescein into a continuous luminal volume, which formed a closed compartment.

The detection of HUVECs during the tubulogenesis process was challenging. After fixation, immunolabeling of isolated trees revealed only a few HUVECs localized on the surface of the epithelium composed of cholangiocytes. Most HUVECs at this stage were left behind on the micropatterned glass surface where they displayed a circular, pilar-like organization (Supplementary Figure S5).

### 2.6. Study of the Self-Organization Process

**Figure 6:**
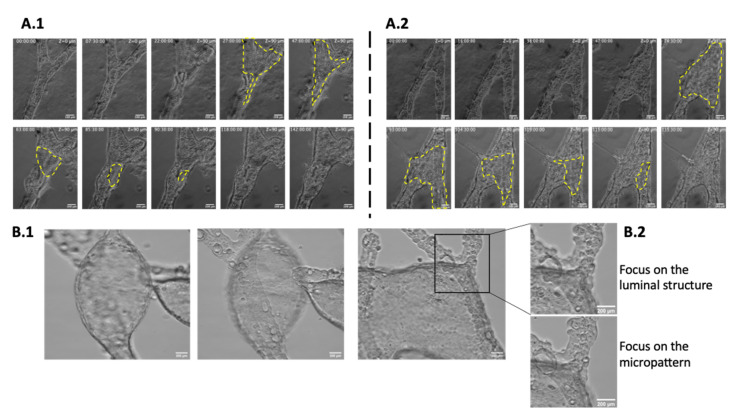
Self-organization process of biliary tubes. (**A**) time-lapse sequence of tubulogenesis along micropatterns: Self-organization of NRCs into a branched tube along a (**A.1**) 45° and (**A.2**) a 30° angle-branched micropattern. The yellow dotted region outlines the edges of the luminal cavity that progressively closes. Scale bars = 100 µm. (**B**) cellular connections along the biliary network: (**B.1**,**B.2**) cell connections formed along the biliary network between the luminal cavities and the underlying cell layer. Insets show a magnification of an anchoring point formed between the 3D luminal structure and the 2D cell layer. Scale bars = 200 µm.

The self-organization process was followed from day 1 after Matrigel addition (day 7 to 8, Figure 1) on two independent experiments via time lapse videos. Supplementary Video S3 (bottom and top) and Supplementary Video S4 (bottom and top) show how the tubular structures develop and Figure 6A.1,A.2 are images extracted at specific time points. 

After analysis, three different steps could be identified: an initial step of about 20 h after Matrigel addition, corresponding to the inward folding of the monolayer. This develops by cell migration into the Matrigel from the edges of the epithelium grown on the collagen coated micropattern (Figure 6A.1 time 0 to 22 h; Figure 6A.2 time 0 to 16 h).

The second step consisted of proliferation of the second layer formed in step 1 into the Matrigel. This was attested by the recruitment of the surrounding ECM, (seen on Figure 6 pictures A.1 T22 to T47h; A.2 T38 to T47h). During this step, this 2nd layer spreaded out of the micropattern, in particular at the level of bifurcations where the epithelium extends between branches. 

During the last three to four days, this second layer progressively folded and closed by a wound-healing like process, enclosing the final luminal structures (Figure 6A.1 T47 to T142h and Figure 6A.2 T74 to T135h Supplementary Figures S6A–C). The luminal structure was formed by the closure of the 2nd layer onto itself, still anchored to the underlying epithelium via cell membrane protrusions. These extended between the 3D structure developed in Matrigel and the epithelium layer grown on the collagen-coated micropatterns (Figure 6B.1,B.2, Supplementary Figure S6D–F). Notably, in fluorescein tests (Supplementary Figure S1 and Supplementary Video S1) the underlying cell monolayer on the micropattern can be seen transparent beneath the interconnected tubular network; where the fluorescent dye has accumulated inside the lumen. Interestingly, this monolayer remains connected to the tubular network, once detached from the micropattern, (Figure 5A,B) where the underlying epithelium, wrapped on itself, can be distinguished through the tubular network.

As a result of the whole process, a tubular network has formed in 3D, guided by the monolayer grown on the pattern, and still attached to it, much like a copy of the micropattern′s geometry.

**Figure 7:**
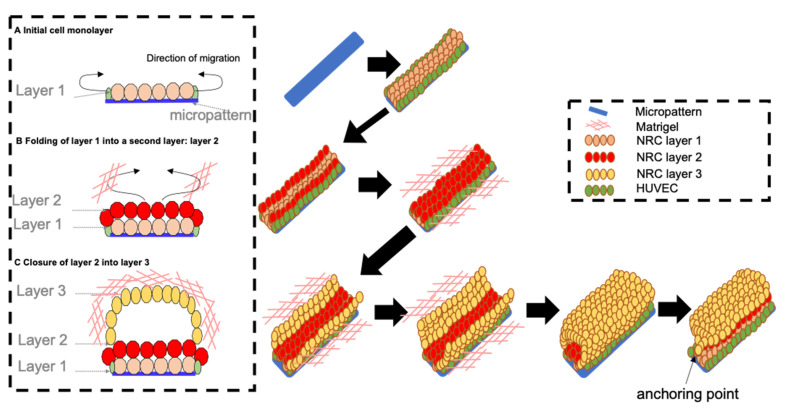
Model for tube formation from micropatterns. (**A**) initial cell adhesion of NRC cells with HUVEC cells on the micropattern. (**B**) folding step of layer 1 into a second layer: layer 2. (**C**) luminogenesis step finishing the self-organization process with the closure of layer 2 into a third layer: layer 3.

On Figure 7 we propose a three-step tentative model of tubulogenesis: 1. control of the 3D network geometry via growth restriction of the biliary epithelium on the HUVEC-pre-seeded micropattern′s geometry. 2. NRC proliferation triggered by Matrigel addition. This provides mechanical support and adhesive cues to the cells, leading to the generation of a second layer over the first cell layer (10 h). This cell layer continues to proliferate and spread in the Matrigel and is probably limited by the tension exerted by the cells firmly adhered to the collagen coating on the micropattern (20 h). This could explain why the final structures follow the pattern, but extend beyond its boundaries, especially at the angles where the epithelium expands between the branches of the pattern. 3. closure of this second layer occurs via a wound-healing-like process (4 to 5 days). A luminal space of interconnected tubes forms with a global geometry that follows the one of the micropattern.

As a result, the tubular network develops in the Matrigel and is connected to the monolayer grown on the micropattern, with a similar geometry (Supplementary Figure S6F) where the hollow tube has developed over a monolayer (referenced as layer 1).

## 3. Discussion

Despite its essential role in draining the bile out of the liver, a bile duct system is still lacking in most bioengineered liver tissues. Bile duct bioconstruction has been hampered by the availability of cholangiocytes, and the difficulty to decipher the properties of the ECM that tune the formation and the maintenance of epithelial tubes.

Reports on the construction of bile tubes are scarce. They are limited to the construction of mono-axial biliary tubes obtained by seeding cholangiocytes on tubular scaffolds made of natural or synthetic hydrogels or PDMS [12,13,14]. These technological feats are promising for pre-clinical or clinical applications, but are limited to dimensions more consistent with the extrahepatic biliary system.

By exploiting the properties of the hepatic extracellular matrix, Shah′s group obtained self-organized branched tubular systems [15] of uncontrolled geometries, or used bioprinting to embed cholangiocytes in branched structures but with diameters in the millimeter range exceeding the dimensions of the intrahepatic trees [16].

We generated self-organized biliary trees on a chip, from micropatterns with geometry and dimensions that emulate the human intrahepatic biliary tree [27]. In addition, the dimensions of the biliary ducts were determined by methodical measurements, assessing the fidelity of the bioengineered ducts to the initial 2D pattern. Our biliary networks retained cholangiocyte specific markers, secretory functions, and were polarized. The trees kept their stable structures over more than 2 weeks and could be easily detached. This represents a versatile system for the integration of other hepatic cell types, or to transfer to fluidic platforms for drug or toxicology studies or in situ implantations in animals.

Finally, we showed that pre-seeding the patterns with endothelial cells significantly expands the luminal area and we provide a model explaining the process of tubulogenesis from a 2D epithelium.

Beyond the interest of our system for the study of biliary functions, its construction by self-organization makes it an interesting model for the study of biliary tubulogenesis. It could also be exploited to investigate the elusive mechanisms of lumen formation and more specific biliary functions such as accumulation and transport of bile as described elsewhere [5]. Moreover, the tubulogenesis process described here appears quite different from that observed in previous work on kidney tubules obtained on 2D micropatterns [20]. In that study, the authors showed that luminogenesis occurs through hollowing on micropattern tube widths up to 15 microns.

In our system, the organogenesis of bile ducts is a two-step process, leading to the formation of an interconnected luminal tree network with branches from 20 to 200 microns wide and several mm long. Interestingly, both steps in our model correspond to the proliferation of a monolayer into the Matrigel and its folding inward to form a tubular hollow structure. This ability of an epithelium to fold inward into a soft extracellular matrix is archetypal of the folding which leads to the formation of the neural tube [30]. In the present case, it seems that the first step of folding leads to an unstable state. Then, the 2nd layer formed during the 1st step proliferates in the Matrigel, ultimately folding inward to form the luminal network. 

The stabilization of the lumen in Matrigel that provides both the adhesive conditions for the polarization of the cells and the mechanical cues allowing the remodeling of the cells conforms to data of the literature on lumen formation conditions [31,32,33]. The fact that the 1st folding does not lead to lumen formation or in a very inefficient way, suggests that the formation of the lumen requires that the cells which form the epithelium have the same matrix environment. Here, the lumen-containing tubes formed within the Matrigel, while the 1st folding that involves cells adhering either on Matrigel or on the colIagen-coated micropattern did not lead to lumen formation nor stabilization. This confirms the results from Bosch et al. of improved tubulogenesis in Matrigel (a laminin rich ECM) when patterns were coated with laminin rather than with fibronectin [20].

This system makes it possible to address these important questions of the boundary conditions that trigger morphogenesis processes like the formation of a lumen.

Following the pioneering work of Bosch, our results confirm that the micropattern approach is effective for epithelial tube engineering. With respect to the previous work, in which the dimensions of the tubes and lumen extent were limited, as it is the case in our protocol with a monoculture of cholangiocytes, we show that the pre-seeding of the micropatterns with endothelial cells is crucial in extending luminal areas. While our preliminary results suggest that their role is more in the order of mechanical support, this question will require further investigation. During embryogenesis, bile ducts develop in the vicinity of the portal vein which probably contributes by the production of soluble factors [34] and extracellular matrix that controls the differentiation and tubulogenesis of cholangiocytes [35]. It is intriguing that the formation of the tubes from a double layer of cholangiocytes facing each other by their apical membrane mirrors the mechanism of formation of the bile tubes from the ductal plate [26]. It will be interesting to understand how this pattern of tubulogenesis is specific to the biliary system, and which specific role the endothelial cells have in the process. This opens the interesting prospect that this protocol, could provide a functional and easily manipulated biliary network for tissue engineering or drug testing. Moreover, we are currently in the process of bioengineering biliary trees from human primary cholangiocytes. However, this has been a challenge due to the instability of these cells to maintain their epithelial phenotype [11], required for the development of 3D biliary networks. Additionally, our preliminary data suggests that our model might allow us to better understand the developmental defects leading to biliary dysgenesis seen in Alagille syndrome or some ciliopathies [36].

## 4. Material and Methods

**Production and Coating of Micropatterns.** Adhesive tree micropatterns with varying line widths and branching angles were printed on PEG-PLL coverslips as described previously [37] (Figure 2A and Supplementary Figure S4A) Micropatterns were coated with 20 μg/mL of collagen I (Collagen type I rat tail, Corning) in phosphate buffered saline (PBS) 1X buffer (Gibco). Seeded cells only adhered to the ECM-coated micropatterns as the PLL-PEG layer is cell-repellent.

**Cell Culture Conditions.** Fluorescent HUVECs-GFP were purchased from Innoprot and maintained according to the provider instructions (Innovative Technologies in Biological Systems, SL, TTFLUOR HUVEC), Spain. NRCs were kindly provided by N.F. LaRusso’s laboratory (Mayo Clinic, Rochester, MN, USA) [29] and maintained as described previously [23]. NRCs and HUVECs-GFP were cultured on collagen I (50 µg/mL) coated flasks. For the co-culture NRCs:HUVEC-GFP, cells were collected and diluted in medium composed of 50% NRC medium and 50% HUVEC-GFP medium (co-culture medium); for the two control conditions, NRCs were collected alone and diluted in the co-culture medium or in the NRC medium. For the co-culture experiments, HUVECs-GFP were first seeded on micropatterns at a density of 1.5 × 10^4^ cells/mL in 2 mL of medium. One day later, NRCs were seeded at a density of 3 × 10^5^ cells/mL in 2 mL of medium. The final co-culture condition mixing NRCs and HUVECs in a 10:1 ratio in the co-culture medium was termed +H in co-culture medium.

**Proliferation Assay.** HUVECs-GFP were seeded at 5 × 10^3^ cells/mL in 150 μL of medium on day 1 in micro-chambers (μ-Slide 8 Well, Ibidi). On day 2, NRCs were added at 5 × 10^4^ cells/mL in 150 μL of medium to the pre-seeded HUVECs-GFP wells or to empty wells. NRC proliferation was assessed in co-culture medium or NRC medium. DIC images were taken from day 1 to day 3 post-NRC seeding and NRC cell density was quantified from the images (see the Image analysis section). To compare the relative proliferation of NRCs in different culture conditions, a ratio of cell densities was used to assess an increase or a decrease in NRC cell proliferation.

**3D Cell Growth Stimulation**. Two to three days after seeding, NRCs formed a confluent monolayer and NRC or co-culture medium containing 10% Matrigel (~0.8–1.2 mg/mL final concentration) (Matrigel Matrix, Basement Membrane, Growth factor reduced, Corning) was added.

**Fluorescein Secretion Test**. After 10 days of culture, the micropatterns were carefully incubated with a 30 μM fluorescein diacetate (FDA)/acetone solution in medium without fetal calf serum (FCS) for 20 min at 37 °C, 5% CO_2_. Following 3 washes with medium without FCS, the samples were ready for image analysis. For the secretion videos (Supplementary Videos S1 and S2), after adding the FDA, no washes were performed, and the trees were immediately imaged.

**Tree Detachment from Micropatterns**. The biliary networks were mechanically detached from the micropatterns. The hydrostatic pressure exerted by the medium ejected from a 200 µL pipettor along the edges of the networks induced the detachment of the trees. Then, the structures were carefully pipetted out and either placed in a 35 mm Petri dish containing the co-culture medium or in an Eppendorf tube placed in ice with 200 μL of Matrigel. The floating tree was further processed for a fluorescein secretion test described in the next section. The Matrigel containing tubular structures was pipetted, using cold pipette tips into a 35 mm dish placed on ice forming a drop on the center of the dish. The dish was incubated at 37 °C for 1 h and then 2 mL of pre-warmed co-culture medium were added. After the tubular networks were detached from the micropatterns, the micropatterns were fixed, permeabilized, and blocked as described in the immunofluorescence section.

**Imaging**

*(a) Differential Interference Contrast (DIC) and Fluorescence Microscopy*. The morphogenesis of biliary networks was monitored via live imaging using a Nikon eclipse TS2R and a Nikon Eclipse TE300 inverted microscopes. To follow the kinetics of fluorescein accumulation in luminal structures, live images were taken after FDA addition in the medium, over 1 h with one image taken every minute.

*(b) Macroscopic Imaging*. Bright field or fluorescent images of tubular networks on micropatterns or detached trees were acquired using an Axio Zoom.v16 (Zeiss) macroscope equipped with the Zen software. For the detached trees, following FDA addition and using a time-lapse plugin, pictures were taken at a rate of 1 picture per min for 30 min.

*(c) Confocal Microscopy*. The geometrical shape of 3D structures formed was examined with a Nikon Eclipse TE-2000-E confocal microscope taking serial images along the Z-axis.

*(d) 3DReconstruction Analysis*. Using the z-stacks taken on the Nikon Eclipse TE-2000-E confocal microscope and the Nikon NIS-elements software, a 3D reconstruction of the tubular structures was generated (see Figure 4K, Supplementary Figure S5A and Supplementary Figure S6F).

**Image Analysis**

*(a) DIC and Fluorescence Microscopy*. For live imaging of fluorescein accumulation in tubular networks on micropatterns and tube formation, movies were reconstituted from fluorescence and DIC images taken from time-lapse experiments: 1 image every min for the fluorescein accumulation and 1 image every 30 min for tube formation with Fiji [38]. For assessing the relative proliferation of NRCs in different culture conditions, a plugins-based customized semi-automatic macro was created to extract the NRC epithelium area using Fiji. DIC images were converted in 8 bits, processed with a background subtraction (30 pixels rolling ball radius), blurred with a Gaussian blur filter (20 pixels radius), manually adjusted in threshold and binarized to extract areas of interest. To compare the relative proliferation of NRCs in different culture conditions, a ratio of cell densities was used to assess an increase or a decrease in cell proliferation.

*(b) Macroscopic Imaging*. Mosaic fluorescence images were stitched using the ZEN Tiles module (Zeiss) and segmented to extract the total area of lumens for each culture condition (mean ± SEM, N = 3 experiments per condition). Fluorescein accumulates in lumens, thus their gray level intensity is higher than in other structures (cells in 2D and cells in 3D but without lumen), which was previously confirmed by confocal analyses with orthogonal views of 3D cell structures after a fluorescein secretion test. Based on this principle, using Fiji [38], a threshold of gray levels was applied on images after background removal (500 pixels rolling ball radius). To extract the luminal area, the threshold was defined using the condition with the lowest organogenesis (NRCs in the NRC medium). A threshold of 32 gray value allowed to extract lumens for this condition. Pixels with lower intensity value were not counted. The same threshold was applied to all images of all conditions. Images were then binarized to measure the global area of white pixels corresponding to lumens. For presentation, fluorescence images have been contrast-enhanced with a minimum gray value = 2 and a maximum gray value = 56.

*(c)Confocal Microscopy*. To estimate the height and width of luminal structures, Z-stacks were analyzed using Fiji with two different plugins-based on a customized semi-automatic macro. The mean height and width were calculated from the average of 2 measurements on the XZ and YZ projections and 2 measurements on the XY projection, respectively. The aspect ratio indicative of the roundness of the tube formed, and the width ratio were calculated. The width ratio defined as the ratio between the tube width divided by branch width of micropattern allowed to evaluate the fidelity of the tube shape to the micropattern geometry. The percentage of lumens was assessed with the orthogonal XZ and YZ views over the set of Z-stacks. If the fluorescein signal was homogeneous and accumulated in the inner part of the volume probed, the structure was classified as lumen. The confocal analyses were performed over the micropattern branches of 100 and 150 μm since for the 10 and 66 μm branches, the organogenesis often occurred out of the micropattern’s width provided. 

**Immunofluorescence.** The micropatterns were fixed (4% formaldehyde, Thermo Fisher 28906 + 5% sucrose, Sigma-Aldrich S0389/PBS) for at least 20 min and permeabilized (0.5% Triton-X 100 (Sigma-Aldrich T8787)/PBS) for 10 min at RT. After 1h incubation with blocking solution containing 0.1% bovine serum albumin (BSA Sigma-Aldrich A2153), 0.2% Triton-X 100, and 0.04% Tween-20 (Sigma-Aldrich, P1379)/PBS, the micropatterns were incubated with primary and then secondary antibodies in blocking solution as described previously [15] (Supplementary Table S1).

**Statistics and Reproducibility** Results are expressed as mean ± SEM. Significance for datasets were calculated in Prism 9 (GraphPad) using a Mann–Whitney test, or a Kruskal–Wallis test and Dunn′s multiple comparison test or with unpaired one-way analysis of variance (ANOVA) and Tukey′s multiple comparison test when comparing more than two conditions. *p* values of statistical significance are represented as **** *p* < 0.0001, *** *p* < 0.001, ** *p* < 0.01, * *p* < 0.05, or not significant if not specified. The number of stacks analyzed are indicated in the figure legends or the Section 4 as well as the number of independent experiments.

## Data Availability

The data presented in this study are available on request from the corresponding authors.

## Supplementary Materials

The following are available online at: https://www.mdpi.com/article/10.3390/bioengineering8080112/s1. Figure S1: Characterization of biliary trees, Figure S2: 3D geometry of biliary networks depending on culture conditions, Figure S3: NRC proliferation in the different culture conditions, Figure S4: Characterization of lumen occurrence and tube geometry as a function of micropattern configuration, Figure S5: Localization of HUVECs on the detached trees and on micropatterns after tree detachment, Figure S6: Epithelial folding upon self-organogenesis, Video S1: Kinetics of fluorescein secretion in a bile duct network, Video S2: A biliary tree detached from a 60° angle-micropattern and secreting fluorescein in its luminal network, Video S3-bottom: Self-organization of NRCs onto a 45° angle-branched micropattern, Video S3-top: Self-organization of NRCs onto a 30° angle-branched micropattern, Video S4-bottom: Self-organization of NRCs with HUVECs onto a 30° angle-branched micropattern., Video S4-top: NRCs self-organization onto a 30° angle-branched micropattern, Table S1: Antibody list.

## Abbreviations

3D	three dimensional
2D	bidimensional
ECM	Extracellular matrix
NRCs or N	Normal Rat Cholangiocytes
HUVECs or H	Human Umbilical Vein Endothelial Cells
FDA	Fluorescein Diacetate
