# Supplementary material for: Self-Organogenesis from 2D Micropatterns to 3D Biomimetic Biliary Trees
Source: Bioengineering (Basel). 2021 Aug 5;8(8):112. doi: 10.3390/bioengineering8080112 (PMC8389215; doi:10.3390/bioengineering8080112)
Supplement: Supplementary file 1 [file bioengineering-08-00112-s001.zip › bioengineering-1285327-Supplementary-final/Final Suppl-Bioeng-3-08-21/bioengineering-1285327-Supplementary-final.pdf]

bioengineering

MDPI

Supplementary Materials

Self-Organogenesis from 2D Micropatterns to 3D Biomimetic Biliary Trees

Emilie Gontran [1,2,†], Lorena Loarca [1,*,†], Cyrille El Kassis [1], Latifa Bouzhir [1], Dmitry Ayollo [3,4,5], Elsa Mazari-Arrighi [3,4,5], Alexandra Fuchs [3,4,5], and Pascale Dupuis-Williams [1,6,*]

1 Université Paris-Saclay, Inserm, Physiopathogenèse et Traitement des Maladies du Foie, F-94800 Villejuif, France; Emilie.GONTRAN@gustaveroussy.fr (E.G.); cyrille.el-kassis@inserm.fr (C.E.K.); latifa.bouzhir@universite-paris-saclay.fr (L.B.)
2 INSERM U-1279, Gustave Roussy, F-94805 Villejuif, France
3 Université de Paris, Inserm, U976 HIPI, F-75006 Paris, France; dmitry.ayollo@gmail.com (D.A.); elsa.mazari@gmail.com (E.M.-A.); Alexandra.FUCHS@cea.fr (A.F.)
4 AP-HP, Hôpital Saint-Louis, 1 Avenue Vellefaux, F-75010 Paris, France
5 CEA, IRIG, F-38000 Grenoble, France
6 ESPCI Paris, Université PSL, F-75005 Paris, France
* Correspondence: lorena.loarca@universite-paris-saclay.fr (L.L) pascale.dupuis-williams@universite-paris-saclay.fr (P.D.-W.); Tel.: +33-1-69-15-79-07
† These authors contributed equally.

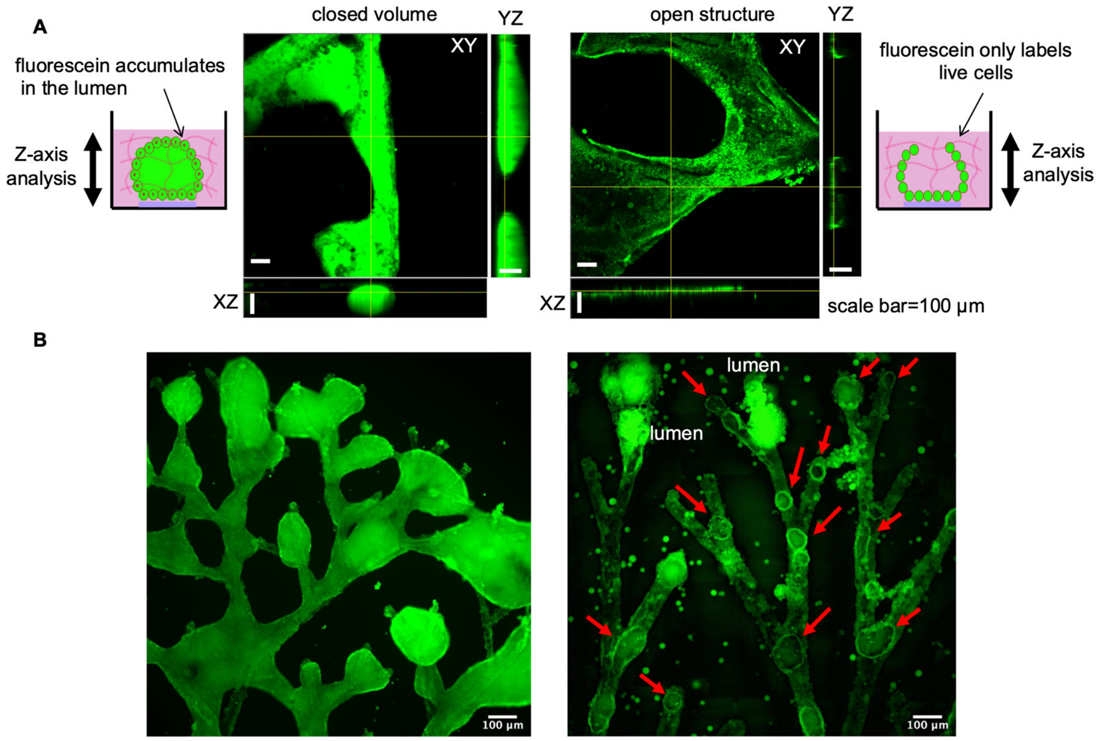

Supplementary Figure S1. Characterization of biliary trees. (**A**) Orthogonal views of biliary tree branches after a Figure 10. (**B**) ratio revealing a continuous luminal network secreting fluorescein (left) *vs* a biliary tree formed in a monoculture of NRCs (right) showing a discontinuous network of lumens annotated “lumen” with several remaining holes in the structure (red arrows).

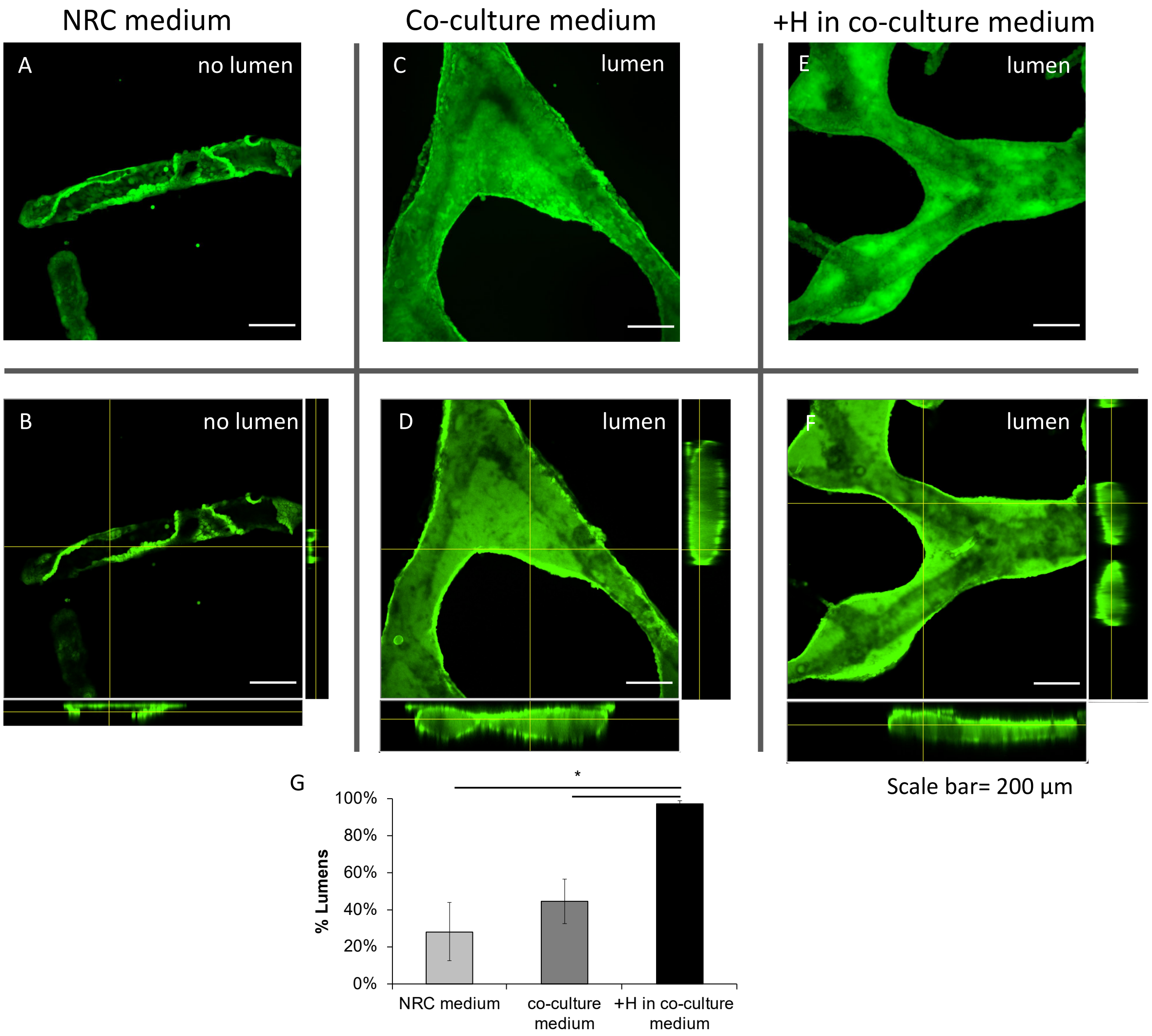

Supplementary Figure S2. 3D geometry of biliary networks depending on culture conditions. (**A**) Z-projection of the sum of slices for a representative biliary structure formed in NRC medium, (**B**) representative slice and corresponding orthogonal views of the same structure shown in (A), (**C**) Z-projection of the sum of slices for a representative biliary structure formed in the co-culture medium, (**D**) representative slice and corresponding orthogonal views of the same structure shown in (C), (**E**) Z-projection of the sum of slices for a representative biliary structure formed in the co-culture medium with HUVECs, (**F**) representative slice and corresponding orthogonal views of the same structure shown in (E). Scale bar=200 µm. (**G**) Mean ± SEM percentage of lumens formed in the different culture conditions. Kruskal-Wallis test with Dunn's multiple comparisons test: NRC medium *vs* +H in co-culture medium: p-value = 0.0238 < 0.05 *; co-culture medium *vs* +H in co-culture medium: p-value = 0.0476 < 0.05 *.

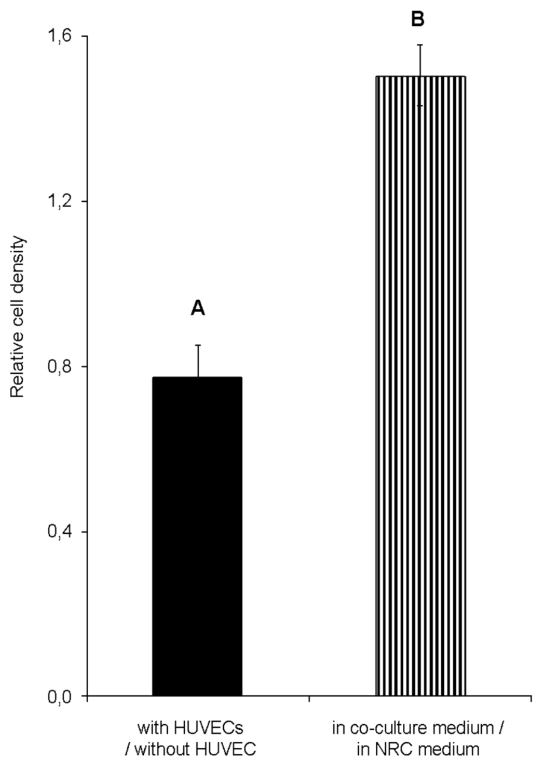

Supplementary Figure S3. NRC proliferation in the different culture conditions. Bar graphs showing relative cell density of (**A**) NRCs with or without HUVECs in the co-culture medium (N = 6), calculated from densities at day 1 post-NRC seeding and (**B**) NRCs in the co-culture medium *vs* in NRC medium (N = 2), calculated from densities at day 4 and day 7 post-NRC seeding.

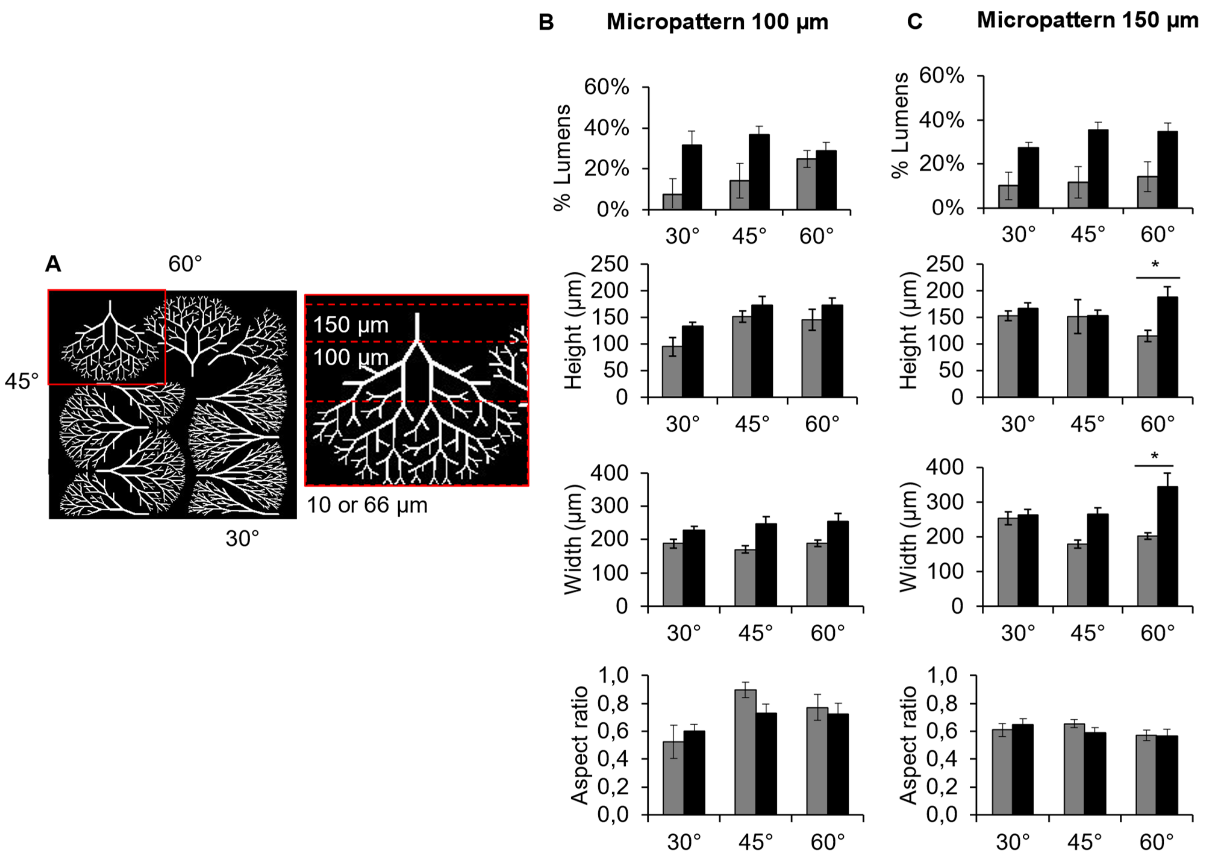

Supplementary Figure S4. Characterization of lumen occurrence and tube geometry as a function of micropattern configuration in the different culture conditions: without HUVEC (grey bars) or with HUVECs (black bars). (**A**) photomask of the micropatterns showing physiologically relevant branch and angle dimensions. Confocal pictures of fluorescein-stained tubular networks were analyzed to calculate height, width, and aspect ratio of luminal structures formed on (**B**) 100 µm-micropattern widths (mean ± SEM), (**C**) 150 µm-micropattern widths (mean ± SEM). Kruskal-Wallis test with Dunn's multiple comparisons test: height 150 µm 60°: $p = 0.0431 < 0.05$ *, width 150 µm 60°: $p = 0.0217 < 0.05$ *.

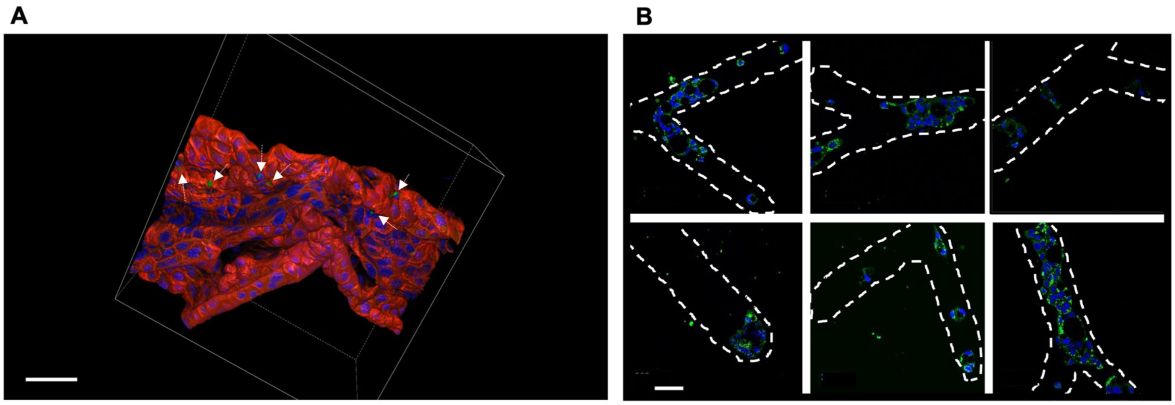

Supplementary Figure S5. Localization of HUVECs on the detached trees and on micropatterns after tree detachment. Biliary networks from NRCs:HUVEC 10:1 co-culture (> 10 days old) were detached. Micropatterns and trees were fixed and probed for F-actin and nuclei (**A**), and nuclei only on (**B**) with the remaining fluorescence of HUVECs-GFP. Confocal pictures showing the positioning of HUVECs-GFP on (A) the detached tree, white arrows (scale bar = 50 µm) and on (B) the micropattern (scale bar = 200 µm).

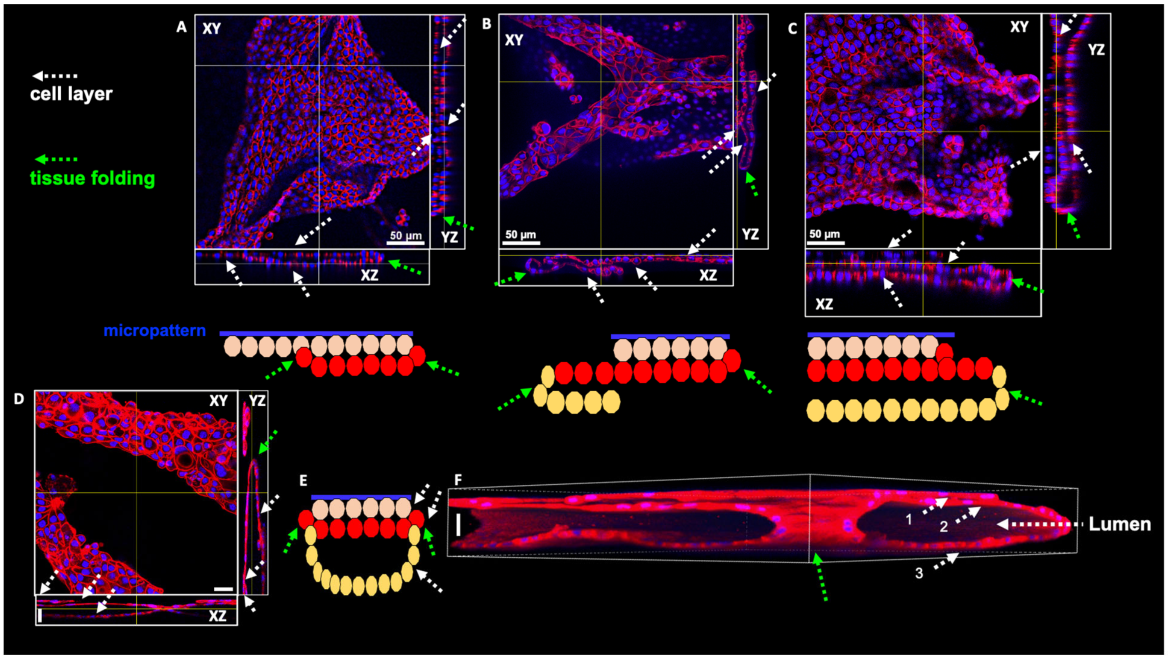

Supplementary Figure S6. Epithelial folding upon self-organogenesis. (**A**–**C**) Longitudinal (XY) and transversal (XZ and YZ) views from immunofluorescence images of biliary structures showing F-actin (phalloidin) and DNA (Dapi). From A to C, successive formation of the first and second layer by spreading of the cell layers into the Matrigel. White arrows indicate the different cell layers identified and green arrows mark tissue folding events. (**D**) Longitudinal and transversal views of a biliary structure showing multiple cell layers and cell sheet folding. (**E**) Corresponding scheme of the cell structure. (**F**) Three-dimensional reconstruction of the biliary network shown in (D) illustrating the 3 superimposed layers forming the biliary network: layer 1 that folds into layer 2 which self-coils into a lumen (layer 3). (D and F): scale bars = 100 µm.

Supplementary Video 1. Kinetics of fluorescein secretion in a bile duct network. Fluorescein accumulation in a tubular network formed in a 10:1 NRCs:HUVEC co-culture in co-culture medium at day 10 post-HUVEC seeding with superimposition of DIC and fluorescence images. Images were captured every minute over one hour. Scale bar = 100 µm.

Supplementary Video 2. A biliary tree detached from a 60° angle-micropattern and secreting fluorescein in its luminal network. One image is taken every minute during 30 min to observe the filling of the lumen with fluorescein. Scale bar = 1 mm.

Supplementary Video 3—bottom. Self-organization of NRCs onto a 45° angle-branched micropattern. NRCs seeded on HUVECs-pre-seeded micropatterns in co-culture medium. An image of a bottom plane of a 45° angle-branched tube is shown. 1 image is taken every 30 min in the plane of the micropattern (named "Z = 0 µm").

Supplementary Video 3—top. Self-organization of NRCs onto a 45° angle-branched micropattern. NRCs seeded on HUVECs-pre-seeded micropatterns in co-culture medium. Image shows an upper plane of a 45° angle-branched tube. 1 image is taken every 30 min at Z position = 90 µm.

Supplementary Video 4—bottom. Self-organization of NRCs with HUVECs onto a 30° angle-branched micropattern. NRCs seeded on HUVECs-pre-seeded micropattern in co-culture medium. Image shows a bottom plane of a 30° angle-branched tube. 1 image is taken every 30 min in the plane of the micropattern (named "Z = 0 µm").

Supplementary Video 4—top. NRCs self-organization onto a 30° angle-branched micropattern. NRCs seeded on HUVECs-pre-seeded micropatterns in co-culture medium. Image shows an upper plane of a 30° angle-branched tube. 1 image is taken every 30 min at Z position = 90 µm.

Supplementary Table S1. Antibody list. Information regarding the primary and secondary antibodies used in the immunofluorescence assays is listed.

Antibody	Catalog number	Company	Dilution
CK7	Sc-23876	Santa Cruz Biotechnology	1:100
CK19	Sc-374192	Santa Cruz Biotechnology	1:100
Epcam	Sc-66020	Santa Cruz Biotechnology	1:10
Osteopontin	Ab-63856	Abcam	1:80
PKCζ	Sc-216	Santa Cruz Biotechnology	1:100
Plakoglobin	13-8500	Invitrogen	1:100
ZO-1		In house made	undiluted
Acetylated α tubulin	5335	Cell Signaling	1:10
Goat anti mouse IgG (H + L) plus 647	A32728	Invitrogen	1:400
Goat anti-rabbit IgG (H + L) 488	A11034	Invitrogen	1:400
Goat anti-rabbit IgG (H + L) 568	A11011	Invitrogen	1:400
Goat anti-rat IgG (H + L) 488	A11006	Invitrogen	1:400
Goat anti-rabbit IgG (H + L) plus 647	A32733	Invitrogen	1:400
Goat anti-mouse IgG_1 (H + L) 568	A21124	Invitrogen	1:400
Goat anti-mouse IgG_{2a} (H + L) 633	A21136	Invitrogen	1:400
Goat anti-mouse IgG_{2b} (H + L) 568	A21144	Invitrogen	1:400
Goat anti-mouse IgG_1 (H + L) 488	A32723	Invitrogen	1:400
